# Vesicular Stomatitis Virus as a Platform for Protease Activity Measurements

**DOI:** 10.1002/cpz1.70062

**Published:** 2024-11-21

**Authors:** Stefanie Rauch, Francesco Costacurta, Dorothee von Laer, Emmanuel Heilmann

**Affiliations:** ^1^ Institute of Virology Medical University of Innsbruck Innsbruck Austria; ^2^ Biological and Environmental Science and Engineering Division King Abdullah University of Science and Technology Thuwal Saudi Arabia; ^3^ These authors contributed equally to this work

**Keywords:** coronaviruses, Mpro, protease inhibitors, SARS‐CoV‐2, vesicular stomatitis virus (VSV)

## Abstract

Protease inhibitors are among the most powerful antiviral drugs. They have been used successfully against viruses, such as the human immunodeficiency virus (HIV), hepatitis C virus (HCV) and severe acute respiratory syndrome coronavirus 2 (SARS‐CoV‐2). Protease inhibitor screening tools are therefore important to identify inhibitors that have the potential to become antiviral drugs. In this article, we describe newly developed cell‐ and virus replicon‐based platforms to screen inhibitors. We developed the methods presented here by genetically modifying vesicular stomatitis virus, a model virus from the family *Rhabdoviridae*. © 2024 The Author(s). Current Protocols published by Wiley Periodicals LLC.

**Basic Protocol**: M^pro^‐On and ‐Off assay

**Alternate Protocol 1**: Virus production with transient P‐ and L TransIT transfection

**Alternate Protocol 2**: Virus production with transient P‐ and L Ca_2_PO_4_ transfection

**Alternate Protocol 3**: Luciferase‐based variation of the On assay

**Alternate Protocol 4**: Screening assay with fluorescence‐activated cell sorting readout

**Support Protocol**: Performing kinetic measurements with Off assay

## INTRODUCTION

**Figure 1:**
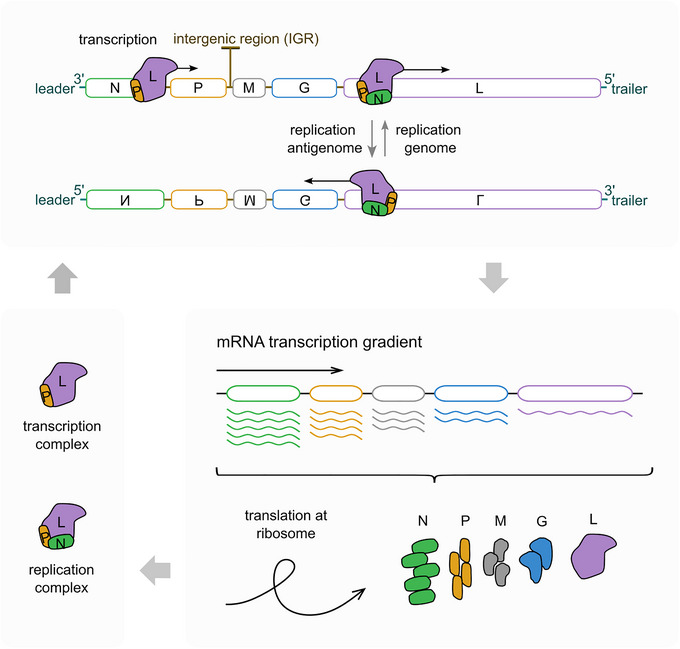
VSV transcription and replication cycle. The intracellular stage of the VSV replication cycle begins by the packaged L‐P complex mediated mRNA transcription. The transcription is guided by the intergenic regions (IGRs) in the VSV genome. These sequences mediate a ∼30% L drop‐off at every gene junction, leading to a transcription gradient from 3′ (N) to 5′ (L). The mRNAs are translated at the ribosome. When enough N are translated and added to the P‐L complex, the transcription switches to replication. During replication, a positive strand (5′ to 3′) antigenome is synthesized as intermediate to the negative strand (3′ to 5′) genome.

Vesicular stomatitis virus (VSV) is a model virus of the family *Rhabdoviridae* and has been widely studied to understand basic virus mechanisms as well as related, non‐segmented negative‐sense RNA genome viruses, such as Ebola, Nipah, and rabies (Pringle & Easton, 1997). In contrast to such viruses, VSV is mostly harmless to humans and its handling only requires biosafety level 2 (BSL‐2) laboratories. Its genome encodes for five proteins, from 3′ to 5′: nucleoprotein (N), phosphoprotein (P), matrix protein (M), glycoprotein (G), and the large protein/polymerase (L). These viral genes are transcribed sequentially by the VSV polymerase in complex with its cofactor P. The intergenic regions (IGRs) between the five genes guide the polymerase in a start‐stop mechanism that leads to a protein gradient from N to L (Abraham & Banerjee, 1973; Ball & White, 1976; Knipe et al., 1975; Schnell et al., 1996). Once enough N is produced, the L‐P‐N complex forms and the viral cycle switches from mRNA transcription to replication. The negative sense genome is transcribed into a positive sense antigenome and vice versa. The negative sense genome is packaged into the viral particle (virion) (Fig. 1).

In assays that measure the expression of a gene, virus spread or, as in this case, protease activity in cell culture, green or red fluorescent proteins (GFP or RFP) are often used to easily visualize the results. However, previous attempts to N‐ or C‐terminally tag VSV proteins with GFP/RFP made them non‐functional, as was shown for protein G. Only in the presence of unmodified G, the C‐terminally tagged GFP‐G virus variant was still able to infect cells (Dalton & Rose, 2001). In case of L, only temporary tags that are removed upon translation by proteases retain the polymerases’ function (Heilmann et al., 2021). To visualize VSVs replication and intracellular localization despite the unfeasibility of N‐ and C‐terminal tags, different groups described intramolecular insertions of fluorescent proteins into P (Das et al., 2006), M (Soh & Whelan, 2015) and L (Heilmann et al., 2019).

We previously leveraged the intramolecular insertion sites for tags in P and L as well as the transient N‐terminal tagging of L to develop proteolytic switches for VSV (Heilmann et al., 2021). We then adapted these switches to serve as proteolytic activity measurement assays (Heilmann et al., 2022).

**Figure 2:**
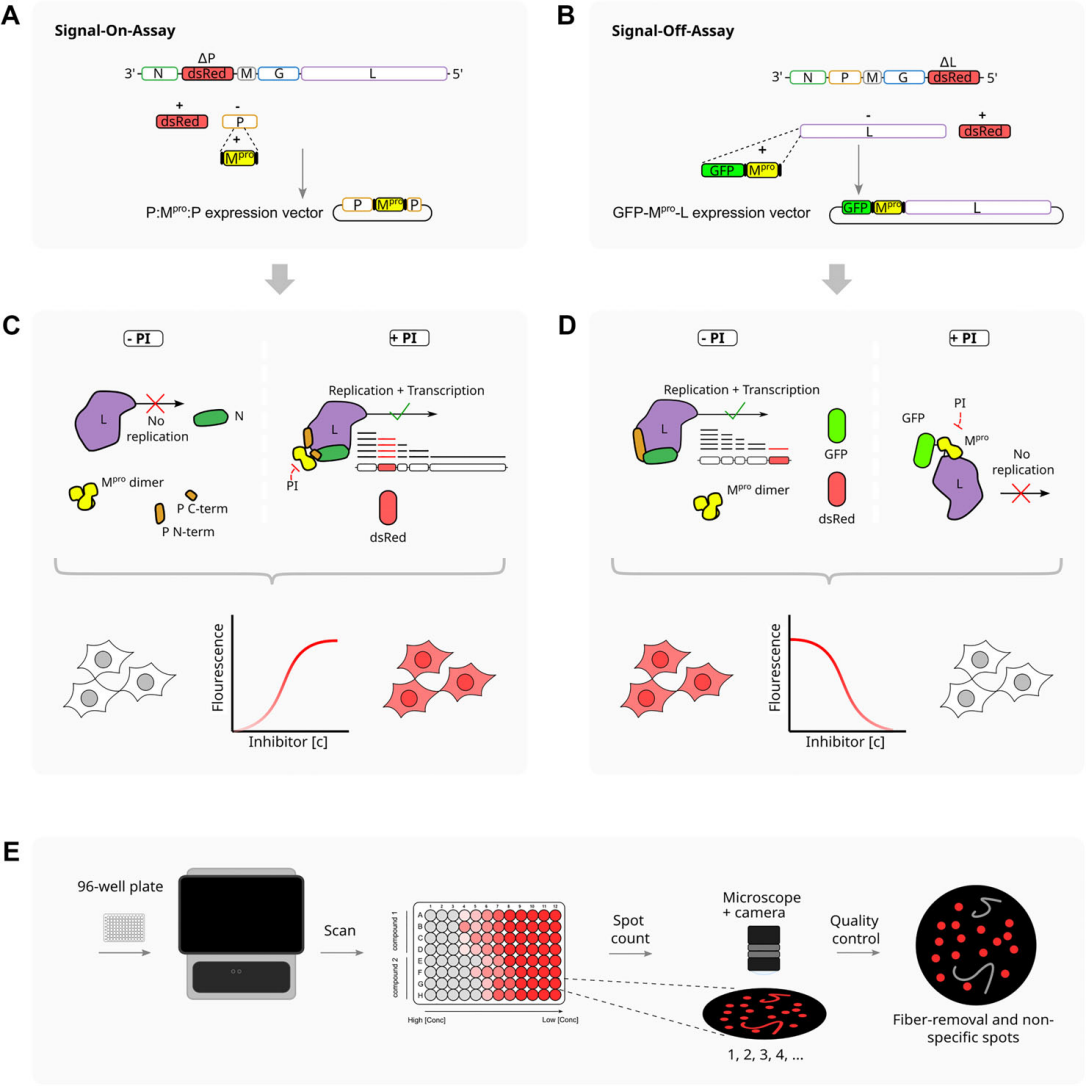
On and Off assays for VSV‐based M^pro^ activity measurement. (**A**) Split of VSV into a replicon and external P:M^pro^:P On‐switch. VSV‐P was replaced with a red fluorescent protein (dsRed), generating VSV‐ΔP‐dsRed. A previously described intramolecular insertion site in VSV‐P was used to insert the main protease (M^pro^) of SARS‐CoV‐2, creating P:M^pro^:P. (**B**) Split of VSV into a replicon and external GFP‐M^pro^‐L Off‐switch. VSV‐L was replaced with a red fluorescent protein (dsRed), generating VSV‐ΔL‐dsRed. A previously described N‐terminal transient tag to L was used to generate GFP‐M^pro^‐L. (**C**) On assay: Cells are transfected with P:M^pro^:P and infected with VSV‐ΔP‐dsRed. In the absence of an inhibitor, M^pro^ degrades. The virus cannot replicate or express red fluorescent protein. Adding an inhibitor saves viral transcription as well as replication. Increasing concentrations lead to red fluorescent protein expression. (**D**) Off assay: Cells are transfected with GFP‐M^pro^‐L and infected with VSV‐ΔL‐dsRed. Without an inhibitor, M^pro^ cleaves GFP‐M^pro^‐L into GFP, M^pro^, and L. L is functional, and the virus can replicate and produce red fluorescence. Adding an inhibitor stops viral replication and red fluorescent protein expression. Green fluorescence is constitutively present in both scenarios. Increasing concentrations lead to red fluorescent protein repression. (**E**) Fluorescent spots (depicted in the plate cartoon as more or less saturated reds, where grey indicates no signal and bright red indicates full signal) correlating with inhibitor concentration and potency are measured with an appropriate high‐throughput fluorescence analyzer. Here, only the schematic representation of the M^pro^‐Off readout is shown, where the dsRed signal decreases at higher concentrations of inhibitor (in the M^pro^‐On, the dsRed signal increases at higher concentrations of inhibitor). Automated as well as manual quality control steps can be performed to remove artefacts such as autofluorescent fibers.

We named the On‐ and Off‐switches in analogy to the well‐known DNA regulatory Tet‐On and ‐Off switches. Hence, the signal produced by the fluorescent marker increases (On‐switch) or decreases (Off‐switch) upon adding a protease inhibitor. A prerequisite to perform the methods described in this article is an established expertise in negative sense RNA virus generation, or more specifically VSV. To generate a functional On‐switch (used in the On assay), we used a variant of VSV, where the P gene was replaced with a red fluorescent protein (dsRed), yielding a replicon depending on the addition of external P (VSV‐ΔP‐dsRed). Then, the previously described intramolecular insertion site in VSV‐P was used to insert a protease of choice. In one instance, this was the main protease (M^pro^) of SARS‐CoV‐2, creating P:M^pro^:P. The split into a VSV replicon and externally expressed P:M^pro^:P switch was done to avoid genetic stability issues of the error‐prone VSV polymerase affecting M^pro^. Furthermore, studying variations of M^pro^, such as mutants, is facilitated by the P:M^pro^:P expression plasmid, which is smaller and therefore easier to modify than a full VSV genome plasmid (Fig. 2A). To generate an equivalent Off‐switch (used in the Off assay), we used a VSV variant in which the viral polymerase or L gene was replaced by the red fluorescent protein dsRed (VSV‐ΔL‐dsRed). External L with a N‐terminal GFP‐M^pro^ tag (GFP‐M^pro^‐L) is expressed from an expression plasmid (Fig. 2B). In the On assay, P:M^pro^:P is transfected into cells, which are then infected with VSV‐ΔP‐dsRed. In the absence of a specific protease inhibitor, M^pro^ cleaves P:M^pro^:P into P‐N‐term, M^pro^, and P‐C‐term. This cleaved protein is not functional, and the virus can neither replicate nor produce red fluorescence. By adding a specific protease inhibitor, P:M^pro^:P remains intact and the viral transcription as well as replication machinery is restored (Fig. 2C).

In the Off assay, GFP‐M^pro^‐L is transfected into cells, which are then infected with VSV‐ΔL‐dsRed. In the absence of a specific protease inhibitor, M^pro^ cleaves GFP‐M^pro^‐L into GFP, M^pro^, and L. The liberated L is functional, the virus can replicate and produce red fluorescent signal. By adding a specific protease inhibitor, the polymerase L remains in the artificial polyprotein GFP‐M^pro^‐L and the viral transcription as well as replication machinery is blocked. GFP is active in both uncut GFP‐M^pro^‐L as well as cut GFP, M^pro^ and L. However, due to rounding up of dead cells, GFP signal will appear brighter in cells actively replicating virus, i.e., wells with low inhibitor (Fig. 2D). Increasing concentrations lead to accumulation of red fluorescence in the On assay (Fig. 2C) and a decrease of fluorescence in the Off assay (Fig. 2D). A high‐throughput fluorescence microscope is used to screen 96‐well plates or similar formats. The plate images are then processed by counting individual spots. In case of a FluoroSpot reader, automated as well as manual quality control steps can be performed to remove artefacts such as autofluorescent fibers (Fig. 2E).

Although the signal strength, consistency, and reproducibility of both On and Off assays are equal when assessing protease activity and inhibition, their characteristics result in each assay being more suitable for certain applications. First, the On or gain‐of‐signal assay is preferable when conducting compound screens. In such a setting, the gain of signal facilitates distinguishing between actual, specific protease inhibition, whereas a loss of signal could also be an artefact caused by the compound killing cells. Similarly, the gain of signal in the On assay might be masked by autofluorescent compounds. However, other than in assays based on purified protease, the autofluorescent signal is clearly distinguishable to the virus‐generated RFP signal, and this difference is assessable via visual inspection with a fluorescence microscope. Depending on the throughput of the screening effort, visual inspection might become impractical. Therefore, we also generated a luciferase‐based On assay, as we expect compound autoluminescence to be exceedingly rare.

The Off assay on the other hand proved more apt to test protease inhibitor resistant mutants, as the signal is easier to quantify in case of a strong resistance. A strong resistance in the On assay sometimes resulted in no signal at all compared to the wild‐type protease, which prevents IC_50_ fold‐change comparison. Furthermore, we use the Off assay to assess the kinetics of proteases and protease mutants.

In the Basic Protocol we describe how to generate stable cell lines to produce viruses necessary to perform On and Off assays, the titration of those viruses, the actual assays and finally how to analyze obtained data. In Alternate Protocols 1 and 2 we detail two alternative means of producing virus, namely transient transfections with Ca_2_PO_4_ and TransIT, respectively. Alternate Protocol 3 describes a luciferase‐based version of the On assay that is particularly useful for large drug screening efforts. If an Fluoro/Immuno/EliSpot device is unavailable, Alternate Protocol 4 describes an alternative readout method based on fluorescence‐activated cell sorting (FACS). Lastly, in the Support Protocol we describe how to use the Off assay to obtain kinetic data of wild‐type proteases in comparison to mutant proteases.

## M^pro^‐ON AND ‐OFF ASSAY

293T cells are seeded and transfected with reporter plasmids, either “On”, i.e., gain of signal or “Off”, i.e., loss of signal, and infected with either VSV‐ΔP‐dsRed or VSV‐ΔL‐dsRed, respectively. After 48 to 84 hr, a FluoroSpot counter or equivalent instrument is used to read red fluorescent spots and determine, e.g., EC_50_s or IC_50_s. For troubleshooting, see Table 1.

**Table 1:** Troubleshooting for Using VSV as Platform for Protease Activity Measurements

Problem	Possible cause	Solution
Too high or low cell count leading to unreliable inter‐assay results	Cell counting not accurate	Count cells with trypan blue in both LUNA II and Neubauer counting chambers (or two other equivalent methods) to obtain more reliable cell counts
Decreasing signal in On assay at high inhibitor concentrations	Cell death due to inhibitor toxicity	Increase cell number for wells, where toxic concentrations are reached, e.g., up to 30 or 40 thousand cells per well
Too low signal	Assay conditions are not yet optimal	Increase the period of transfection (e.g., from 8 hr to overnight); use more DNA for transfection; use fewer cells for transfection; increase MOI from 0.1‐0.2 or re‐titrate virus to confirm titer
Positive signal in all wells, no distinction between inhibitor negative/positive wells	MOI too high	At high MOIs, the virus will kill all cells and produce fluorescent signals; lower the MOI, re‐titrate the virus or check whether MOI calculation was faulty
Excessive or no precipitate after Ca_2_PO_4_ transfection	pH of 2× HEPES/HBS buffer was inaccurate	Measure buffer concentration again and make sure it is adjusted to pH 7.1

The assays described in this protocol require replication incompetent VSV variants missing either L or P genes. A prerequisite to perform this method is to have a setup for VSV rescues established and the viruses available that are described in the following protocols. VSV‐∆P‐dsRed or luciferase are replication deficient single‐cycle VSVs where the P gene is exchanged by dsRed (Muik et al., 2012) (see Supporting Information, supplemental sequence 1 and a description of all sequences in Table 2) or firefly luciferase (Heilmann et al., 2022) (see Supporting Information, supplemental sequence 2). These viruses can be produced on cells expressing VSV‐P (see Supporting Information, supplemental sequence 3). VSV‐∆L‐dsRed is a replication deficient single‐cycle VSV, where the L gene is exchanged by dsRed (Muik et al., 2012) (see Supporting Information, supplemental sequence 4). This virus can be produced on cells expressing VSV‐L (see Supporting Information, supplemental sequence 5). To produce both ‐∆L and ‐∆P viruses, transiently transfected or stable cell lines can be used.

**Table 2:** Plasmid Sequences Provided in the GenBank (.gb) Format, a Standard Format that Includes Sequence Annotations

Supplemental sequence no.	Name	Description
1	VSV‐∆P‐dsRed	VSV variant with P replaced by dsRed
2	VSV‐∆P‐luciferase	VSV variant with P replaced by luciferase
3	VSV‐P	VSV phosphoprotein gene
4	VSV‐∆L‐dsRed	VSV variant with L replaced by dsRed
5	VSV‐L	VSV polymerase gene
6	VSV‐G	VSV glycoprotein gene
7	Gag‐Pro‐Pol	HIV Gag‐Pro‐Pol genes
8	M^pro^‐On	VSV phosphoprotein gene with intramolecular M^pro^ insertion
9	M^pro^‐Off	VSV polymerase gene with N‐terminal GFP‐M^pro^ tag

### Materials

HEK 293T (293tsA1609neo, ATCC, cat. no. CRL‐3216) or equivalent cells
Dulbecco's modified Eagle medium (DMEM), supplemented (see recipe)
Lentiviral vector plasmids VSV‐P or ‐L (see Supporting Information, supplemental sequences 3 and 5)
VSV‐G (see Supporting Information, supplemental sequence 6)
HIV Gag‐Pro‐Pol (see Supporting Information, supplemental sequence 7)
Optimem (Gibco, cat. no. A4124801, or equivalent)
TransIT‐LT1 or ‐PRO reagent (MirusBIO, cat. nos. MIR 2300 or MIR 5700, respectively) or equivalent transfection reagent
BHK‐21 (ATCC, cat. no. CCL‐10) or equivalent cells
Trypsin (Gibco, cat. no. 12604‐021, or equivalent)
10 mg/ml polybrene (Sigma, cat. no. TR‐1003‐G, or equivalent)
M^pro^‐On (see Supporting Information, supplemental sequence 8)
M^pro^‐Off (see Supporting Information, supplemental sequence 9)
10 mg/ml blasticidin (Invivogen, cat. no. ant‐bl‐05, or equivalent)
50 mg/ml hygromycin (Thermofisher, cat. no. 10687010, or equivalent)
VSV‐∆P‐dsRed (see Supporting Information, supplemental sequence 1)
VSV‐∆L‐dsRed (see Supporting Information, supplemental sequence 4)
Phosphate‐buffered saline (PBS) (Gibco, cat. no. 10010023, or equivalent)
20% saccharose solution (see recipe)
100% ethanol
FluoroBrite DMEM, supplemented (see recipe)

Incubator, 37°C, 5% CO_2_
Vortexer
6‐ and 96‐well tissue culture plates
1.5‐ and 2‐ml microcentrifuge tubes
Water bath
1.5‐, 5‐, 15‐, and 50‐ml sterile reaction tubes
Centrifuge suitable for FACS tubes/standard centrifuge tubes (e.g., Hettich Rotana 460R)
T25, T75, T125 flasks or 10‐ to 15‐cm dishes
0.45‐µm filter
5‐, 15‐, and 50‐ml centrifuge tubes
5‐, 10‐, and 25‐ml sterile pipettes
Biosafety cabinet class 2 (BSL‐2)
Tissues
500‐µl, 1.1‐ and 2.2‐ml deep‐well plates for dilutions
Mono‐, 8‐ and/or 12‐channel pipettes, volume ranges 5 to 100, 30 to 300, 100 to 1000 µl, or equivalent
Medium pools/containers/solution basin (BioRad, cat. no. ML10542, or equivalent)
Vacuum pump
LUNA II cell counter, Neubauer counting chamber, or equivalent device
Microscope and fluorescence microscope
Wet chamber, i.e., plastic box with water‐soaked paper towel
Fluoro/Immuno/EliSpot counter or equivalent measurement system (for fluorescence) with provided CTL software
GraphPad Prism software

### Virus production with stable P‐ and L‐lentiviral transduction

Stable cell lines facilitate repeated virus production and maximum scalability. For stable transduction, lentivirus particles are generated and then used to infect a cell line of choice, e.g., HEK 293T cells. For the safe use of lentivirus particles, 3‐plasmid systems are a state‐of‐the‐art method. One plasmid contains VSV‐G with a standard promoter (see Supporting Information, supplemental sequence 6) as its glycoprotein has a broad tropism and particles with VSV‐G on the surface will infect a wide range of cell lines. The second plasmid contains HIV Gag‐Pro‐Pol (see Supporting Information, supplemental sequence 7) for reverse transcription and integration enzymes. The third plasmid contains the lentiviral elements necessary for the HIV enzymes to recognize the sequence of stable transduction. VSV‐P/L and M^pro^‐On/Off used in this protocol are lentiviral plasmids based on pLenti CMVie‐IRES‐BlastR (Addgene, accession #119863) with recognition sequences for lentiviral packaging, reverse transcription, and integration. All four can be used to generate lentiviruses. Any transfection method can be used to produce lentiviral particles. TransIT is a convenient method for this purpose and is therefore described below.

1 Seed 2 × 10^5^ 293T cells per well in a 6‐well plate, incubate overnight in fully supplemented DMEM at 37°C, 5% CO_2_.
2 Transfect cells as described in further detail in M^pro^‐On and ‐Off protocol steps (steps 44 to 49), with either P or L and add VSV‐G and Gag‐Pro‐Pol to the transfection mix.
3 For one 6‐well plate, prepare the plasmids below and mix with vortexer:
  12.5 µg Gag‐Pro‐Pol
  1 µg VSV‐G.
  The final volume may vary depending on the starting concentration of the stock constructs.
4 Prepare as many microcentrifuge tubes as there are wells to be transfected with 200 µl DMEM medium without additives (or Optimem).
5 Add 1/6^th^ of the Gag‐Pro‐Pol/VSV‐G mix to each microcentrifuge tube.
6 Add 1 µg of the desired lentiviral construct to one microcentrifuge tube, e.g., VSV‐P.
7 To control transfection efficiency, a fluorescent plasmid can be transfected in parallel in one of the 6 wells.
8 Add 9 µl TransIT‐LT1 transfection reagent (∼3× as much as µg DNA) or 3 µl TransIT‐PRO plus 1.5 µl booster per tube.
9 Incubate at room temperature for 20 min.
10 Add previously prepared transfection mixes (200 µl medium/Gag‐Pro‐Pol/VSV‐G/lentiviral vector) to individual wells of the 6‐well plate containing 293T cells.
11 Collect supernatants from each well after 24, 48, and 72 hr and store them in appropriate vessels (e.g., 1.5‐, 5‐, 15‐, or 50‐ml sterile reaction tubes). Refill well with fresh, medium prewarmed in a water bath. Either store and pool supernatants or transduce new 293T, BHK‐21, or any cell line of choice.
12 For transduction, trypsinize 293T cells and seed 2 × 10^5^ per well in a 6‐well plate. Seed a plate for each construct.
13 Add a range of volumes from collected supernatants, e.g., 50, 100, 200, 400, and 800 µl in different wells.
14 Add 8 µg/ml polybrene to improve lentiviral cell penetrance.
15 Centrifuge the 6‐well plate 1 hr at 1000 × *g*, 37°C (so called “spinfection”).
16 Incubate cells until they are dense enough to split. Cells with high lentiviral load might experience cell death.
17 After 2 to 3 days, split cells and add a selective marker. VSV‐P and M^pro^‐On encode the hygromycin resistance cassette, VSV‐L and M^pro^‐Off encode a blasticidin resistance cassette.
18 Add 10 µg/ml blasticidin for 293T and 12 µg/ml for BHK21, or 400 µg/ml hygromycin for 293T and 600 µg/ml for BHK‐21.
19 Add selective marker to untransduced cells as killing control.
  If the toxic dose for the chosen cell line is unclear, a dose‐response can be performed before the transduction on wild‐type cells.
20 After several days, non‐transduced cells have died, whereas transduced cells survived. Grow these cells up in larger culturing vessels, e.g., T25, T75, T125 flasks or 10‐ to 15‐cm dishes, while maintaining selection medium.
21 Establish a cell bank in liquid nitrogen for future use.
22 Seed stably expressing cells in 10‐ or 15‐cm dishes with 5 or 10 million cells per dish, respectively. Use 5 to 10 ml in a 10‐cm dish and 15 to 20 ml in a 15‐cm dish. Use as many dishes required to obtain at least 45 ml volume or multiples of that (e.g., 90 ml, 135 ml, etc.).
  The more dishes are seeded here and later infected, the bigger the virus stock yield will be.
23 The next day, infect cells with a multiplicity of infection (MOI) of 0.01 of the appropriate virus (P expressing cells for VSV‐ΔP‐dsRed, L expressing cells for VSV‐ΔL‐dsRed). Use Supplemental File 1 (see Supporting Information and description in Table 3) as MOI calculation aid.

**Table 3:** Calculation Aids: Two Excel Sheets in the .xlsx Format are Provided to Facilitate the Described Protocols

Supporting Information file	Name	Description
Supplemental file 1	Virus titration sheet	A printable template for counting infected and non‐infected wells as well as a formula sheet to calculate viral titers according to the method of Spearman and Kärber
Supplemental file 2	Assay calculation sheet	A sheet to facilitate calculating amounts of cells, virus, and inhibitor in the assays
Supplemental file 3	Raw data file	Collection of all the raw data used to produce the graphs shown in Figure 7

24 Wait up to 3 days until all cells are dead and strong red fluorescence is visible.
25 Collect supernatant while filtering with a 0.45‐µm filter.
26 Either freeze the supernatant directly or proceed to concentrate the virus.
27 To concentrate the virus, add 45 ml of supernatant to a 50‐ml tube. If exactly 45 ml are unavailable or some medium has evaporated, fill up to 45 ml with PBS.
28 Prepare a sterile filtered 20% saccharose solution.
29 Carefully underlay the 20% saccharose below the supernatant with a 5‐ or 10‐ml pipette.
  There should be a clear phase separation between the saccharose cushion and the supernatant.
30 Centrifuge the 50‐ml tube overnight at ≥4600 × *g*, 4°C.
31 The next day, prepare an ethanol‐soaked tissue below a BSL‐2 laminar flow.
32 Carefully discard the supernatant and turn the Falcon tube upside down onto the ethanol‐soaked paper.
33 Remove any rest of medium by tapping the Falcon tube onto the soaked paper.
34 Resuspend the viral pellet, which typically is barely visible if at all, with 100 to 400 µl of medium.
35 Produce aliquots and freeze them at −80°C.

### Virus titration with TCID_50_

36 Seed 1000 cells stably expressing either P or L in 100 µl per well in 96‐well plates 1 day before infection. Transiently transfected cells can also be used but increase the cell count to at least 5000 in this case.
37 Prepare serial dilutions of the virus in either logarithmic (1:10^1^ = 1:10) or half‐logarithmic (1:10^0.5^ = 1:3.162) dilution steps in a deep‐well plate. Logarithmic dilutions can be done, e.g., with 900 µl + 100 µl, whereas half‐logarithmic dilutions can be done, e.g., with 900 µl + 416 µl.
38 Transfer the volume from the deep well plate to eight 96‐well plates with an 8‐ or 12‐channel pipette. With an 8‐channel pipette, start from the highest dilution, i.e., the lowest amount of virus on the right side of the plate to be able to reuse the tips.
39 Incubate for up to 6 days at 37°C.
40 Print template sheet provided in Supplemental File 1 (see Supporting Information and description in Table 3) and read out cell death.
  Medium containing phenol red is helpful to concentrate on the wells where cell death occurred and expedite the counting. Wells, where the cells did not die, will overgrow and phenol‐red will shift from red to yellow (see Supporting Information, Supplementary Figure 1). Fluorescence can also be used to determine positive and negative cells with more precision.
41 Fill out dead and alive well count in Supplemental File 1 to retrieve viral titers according to the following formula:
$$\frac{TCID^{50}}{ml}=x\;\times 10^{\left[k+d\;\times \;\left(0.5-\left(\frac{r}{n}\right)\right)\right]}$$
  where, k is the positive exponent of the highest dilution tested (e.g., highest dilution tested = 10^−11^; k = 11); r is the total number of wells that were infected, but cytopathic effect negative (not including control wells without virus); x is the extrapolating factor to get TCID_50_ per ml (x = 10 when assay is performed according to this protocol, i.e., 100 µl virus dilution is added by well; d is the spacing between dilutions (d = 0.5 for half‐logarithmic dilutions; d = 1 for 10‐fold serial dilutions); and n is the number of wells per dilution (n = 8 when assay is performed according to this protocol).

### M^pro^‐On and ‐Off assay

#### Day 1: Seeding of HEK 293T cells

42 Grow HEK 293T cells in T25, T75, T170 flasks, 10‐ or 15‐cm dishes, or equivalent vessels.
43a Seed 3–4 × 10^5^ HEK 293T cells per well of a 6‐well plate 1 day before transfection if transfection and treatment are both done on the same day.
43b Seed 2–3 × 10^5^ HEK 293T per well of a 6‐well plate if transfection is on the next day and treatment is 1 day after the transfection (i.e., overnight incubation).
  The cell density might vary according to the counting method.

#### Day 2: Transfection of cells

44 Transfect plasmids with TransIT‐LT1 transfection kit or TransIT‐PRO with 1000 ng plasmid DNA of M^pro^‐On (see Supporting Information, supplemental sequence 8) or M^pro^‐Off (see Supporting Information, supplemental sequence 9).
  - With TransIT‐LT1, the recommended ratio of µg of DNA to µl of reagent is 1:3 (e.g., 1 µg DNA + 3 µl TransIT reagent).
  - When using TransIT‐PRO, a 1:1 ratio of µg of DNA and µl of transfection reagent (white/transparent vial) is recommended by the manufacturer (i.e., 1 µg + 1 µl TransIT‐PRO). Additionally, transfection booster (brown vial) is added at half of the TransIT reagent volume (i.e., 2 µl TransIT‐PRO reagent + 1 µl booster).
45 Adjust the volume of reaction medium (it is important that medium must not contain additives, e.g., FCS or pen/strep; i.e., Optimem or DMEM without supplements) to the amount of DNA (i.e., 2000 ng = 2 µl TransIT‐PRO, 1 µl booster = 200 µl medium). 1 µg of DNA is recommended by the manufacturer to be mixed in 100 µl medium. Consult Mirus TransIT manual for more detailed instructions.
46 Incubate DNA/TransIT reagent/medium mixture for 20 min at room temperature.
47 Add mix to cells in 6‐well plate. Tilt the 6‐well plate so that the medium accumulates in one corner and adding the mix does not detach the cells.
48 Gently move the 6‐well plate to distribute transfection mix in the medium.
49 Incubate at 37°C for 8 to 10 hr or overnight.

#### Day 2 or 3: Infection and treatment of cells

**Figure 3:**
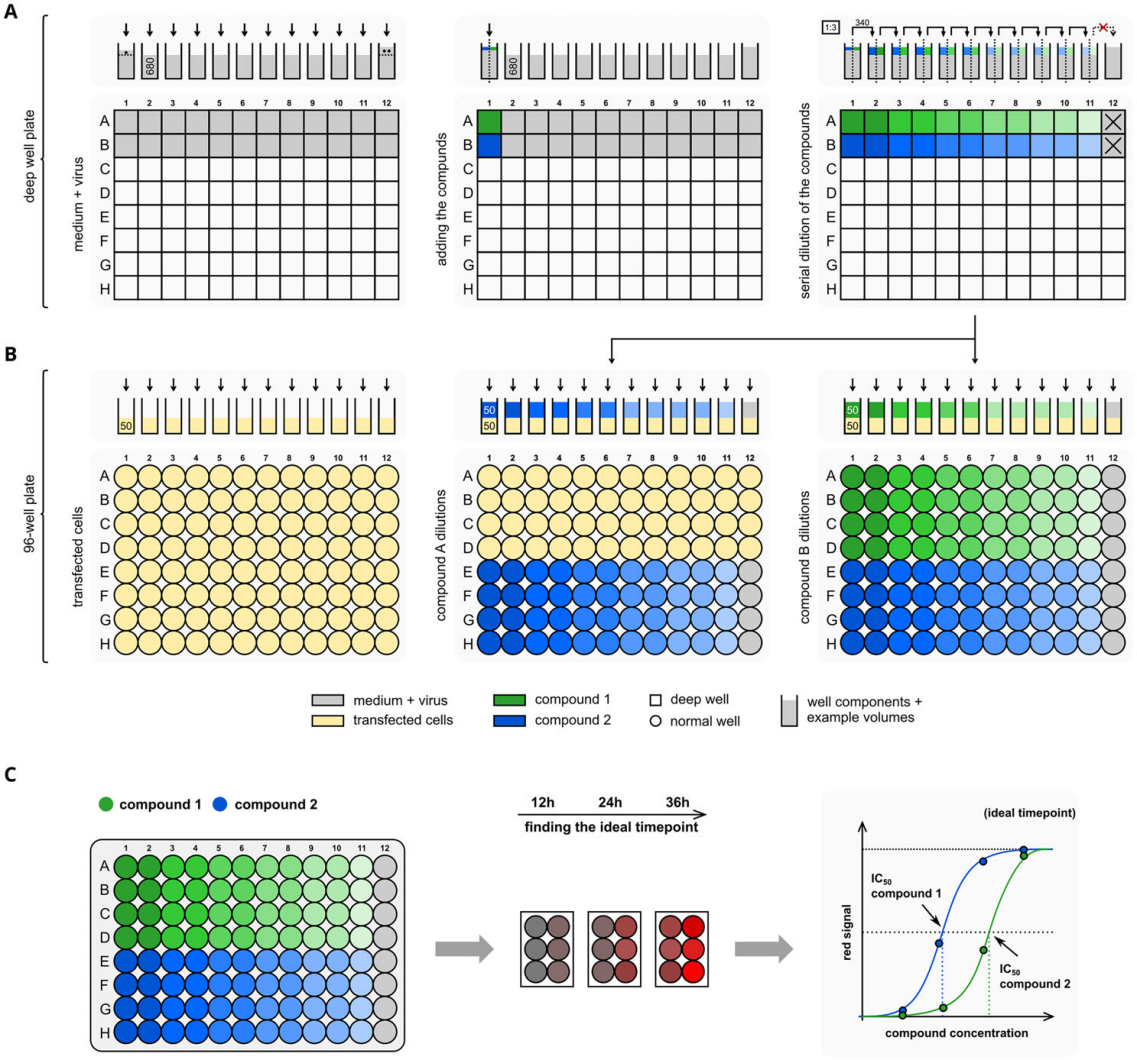
Schematic visualization of the compound + virus mix and serial dilutions. (**A**) Serial dilution of compound + virus mix in a deep‐well plate. Appropriate volumes of virus solution of desired MOI are added to the deep‐well plate. Compounds are added at the highest concentration (double of the highest tested concentration) in the first column (1) of the plate. Subsequently, compounds are serially diluted until column 11 reaching the lowest tested concentration. Column 12 is kept as no‐inhibitor control. (**B**) Transfer of the compound + virus mix to the experimental plate (regular flat bottom 96–well plate) where cells where previously seeded. Proceed by adding 50 µl of compound + virus mix into each row of the plate (e.g., in quadruplicates). (**C**) Determining IC_50_s of different inhibitors in exemplary On assay. Optimal timepoints for data collection can be found by repeated scanning in different time intervals. IC_50_’s are determined with Graphpad Prism or equivalent software.

50 8 to 10 hr after transfection (or after overnight incubation), detach HEK 293T cells with trypsin from the 6 well‐plates.
51 To detach cells, remove the supernatant using a vacuum pump or pipette.
52 Add 400 µl of 1:1 trypsin:PBS solution and place in the incubator at 37°C for up to 5 min.
53 Tap and move 6‐well plate until cells are detached and single in solution.
54 Add 600 µl of standard DMEM (with supplements) or DMEM without phenol red (e.g., FluoroBrite).
55 Pipet off 1 ml, resuspend the cells and transfer to a 2‐ or 5‐ml microcentrifuge tube or 15‐ml Falcon tube.
  If the experiment requires transfecting more wells with the same construct, it is possible to pool the cell suspensions from different wells in one larger tube or a 15‐ml Falcon tube.
56 Add 500 µl of standard DMEM (with supplements) or DMEM without phenol red (e.g., FluoroBrite) to harvest remaining cells.
57 Pipet 500 µl of cell suspension to the same tube containing previously harvested cells.
58 Count and seed 1−2 × 10^4^ cells/well in 50 µl medium/well, i.e., 2−4 × 10^5^ cells/ml in a 96‐well plate. Use convenient medium pools, containers, or solution basins and multichannel pipets for seeding.
59 For virus and compound dilutions, prepare the virus solution to an MOI of 0.1. For “On” or gain‐of‐signal assay, use VSV‐∆P‐dsRed (see Supporting Information, supplemental sequence 1) or luciferase (see Supporting Information, supplemental sequence 2). For “Off” or loss‐of‐signal assay, use VSV‐∆L‐dsRed (see Supporting Information, supplemental sequence 4).
60 Prepare compound + virus mix dilutions. An Excel spreadsheet can be used to calculate both the amount of virus and serial dilution volumes (see Supporting Information, Supplemental File 2). Alternatively, webtools (e.g., https://www.aatbio.com/tools/serial‐dilution) can also be used to calculate virus MOIs and inhibitor dilutions.
61 Distribute the virus solution to a deep‐well plate (for graphical visualization see Figure 3). Depending on the number of samples to test, use a deep‐well plate that fits your volume needs, e.g., 500‐, 1000‐, or 2000‐µl.

62 Serially dilute with single‐ or multichannel pipets. Make sure to change tips at each dilution step.
63 Add 50 µl of compound + virus (MOI 0.1) mixture to each well to reach desired concentrations.
  Prepare twice the desired compound concentration, since it will be diluted 1:1. The virus MOI does not change by adding it to the well in a 50:50 µl:µl ratio, since MOI depends on the absolute number of cells and viruses.
64 After the addition of all the experiment components, check under the microscope if cells are evenly distributed in the well. If they are unevenly distributed, tap each side of the plate(s) gently 3 to 4 times and check again under the microscope. When cells are evenly distributed, place the plates into a plastic box with a water‐soaked paper towel (wet chamber).

### Screening assay with FluoroSpot readout

**Figure 4:**
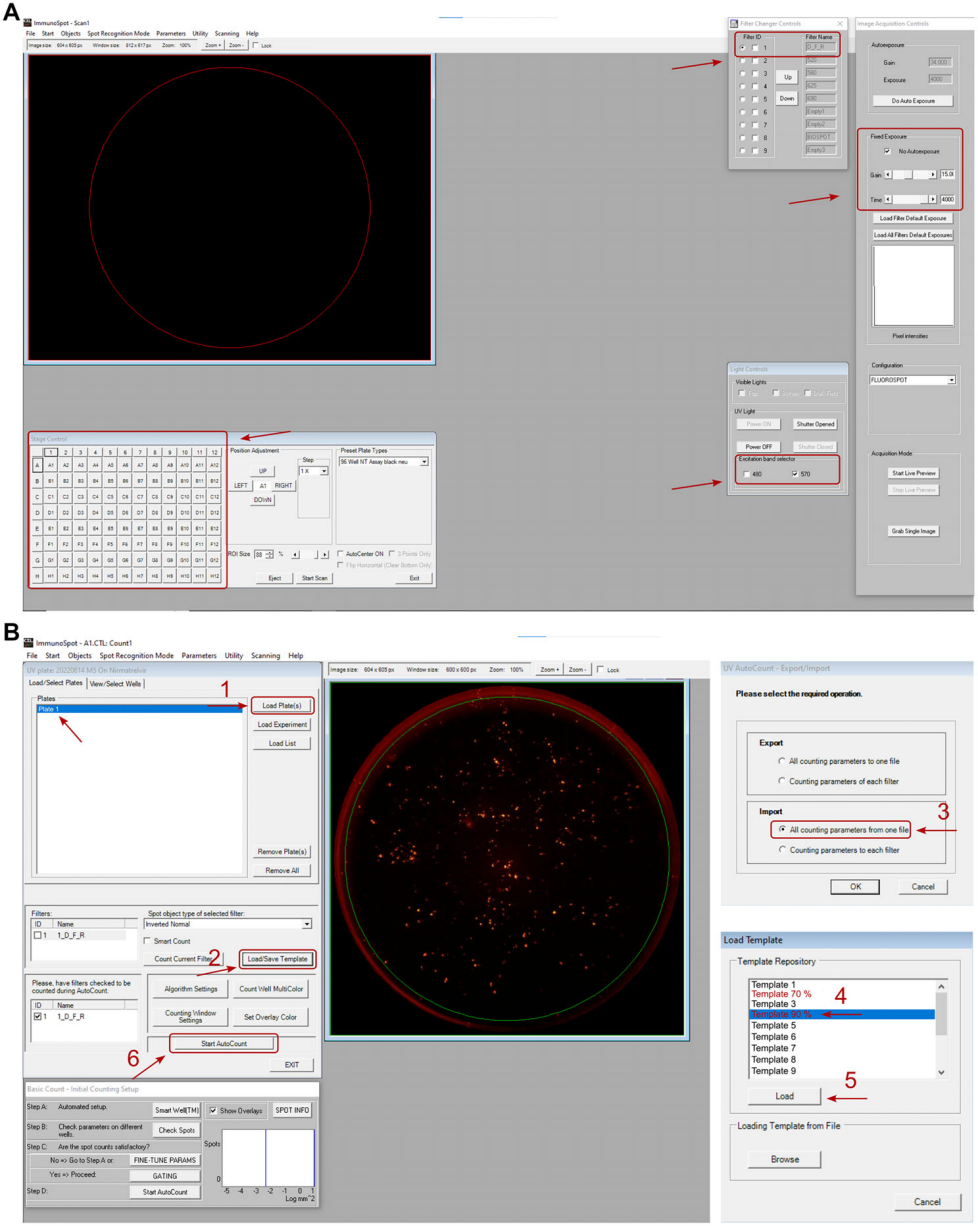
CTL FluoroSpot X Suite for plate scanning and spot counting. (**A**) Scan suite. Stage control, 96‐well control panel; filter change controls, filter ID 1 D_F_R; light controls, only 570 nm; image acquisition controls, no autoexposure; gain, 15.00; time, 4000. (**B**) Counting suite: (1) load plate(s), select the plate from the previous scan; (2) load/save template; (3) import, all counting parameters from one file; (4) select the template; (5) load; (6) start AutoCount.

65 After 48 to 84 hr, check fluorescence signal with a fluorescence microscope. If the signal intensity is sufficient, remove supernatant (if medium contains phenol red) or leave medium (if transparent medium was used) and count fluorescent spots (individual fluorescent cells or cell clumps).
  Depending on the camera of the microscope, phenol red does not interfere with screening. Therefore, assess the available set up with test plates a priori to determine whether medium without phenol red is necessary.
66 Spots (fluorescent cells or clumps) can be counted in a Fluoro/Immuno/EliSpot or equivalent device.
67 When using a Fluoro/Immuno/EliSpot with the manufacturer‐provided software CTL switchboard, scan 90% (if the medium is removed) or 70% (if the medium is not removed) of each well area concentrically to exclude reflection from the well edges and normalize to the full area. Apply automatic fiber exclusion while scanning.
68 The excitation wavelength for dsRed/RFP is 558 nm and the emission peaks at approx. 580 nm; use the D_F_R triple band filter to collect fluorescence (Fig. 4A).

69 Fluorescent cells will be visible as spots on the well images saved by the high‐throughput microscope. Count those spots by customizing the parameters in the software according to preferences for sensitivity, spot size, background and additional parameters. The preferences can be saved as counting templates, which after being defined once are available to be imported before every run (Fig. 4B).
70 Alternatively, spot counts can be performed with an equivalent counter, such as BZ‐X810 All‐in‐One fluorescence microscope from Keyence, a Cytation 1 imaging reader from BioTek, or any equivalent device.
71 When using a Fluoro/Immuno/EliSpot counter, make use of the software‐integrated quality control steps like manual fiber removal after counting.

### Data analysis

72 CTL Fluoro/Immuno/EliSpot software provides Excel sheets containing data. Depending on the reader or FACS software, different export options might be available.
73 Optional: A normalization step can be performed (i.e., max normalization or min‐max normalization).
74 Data can be analyzed using GraphPad Prism (any version).
75 When using GraphPad Prism, to calculate the IC_50_ values, perform a non‐linear regression analysis with the following equation (sigmoidal, 4 parameter logistic):
$$Y=B+\;\frac{T-B}{1+{\left(\frac{IC_{50}}{X}\right)}^{HillSlope}}$$
  where, T is the top of the curve (plateau); B is the bottom of the curve; X is the concentration; and HillSlope is the steepness of the curve when the signal is increasing (3CL^pro^/M^pro^‐On) or decreasing (3CL^pro^/M^pro^‐Off).
76 Optionally, check slope curves and confine slope steepness to avoid over‐fitting. Off‐assay curves have negative slopes, i.e., the confinement could be –3; On‐assay curves have positive slopes, i.e., the confinement could be +3.

## Alternate Protocol 1 VIRUS PRODUCTION WITH TRANSIENT P‐ AND L TransIT TRANSFECTION

If the means for the generation of stable cell lines, such as the required plasmids, are not available, virus can also be produced on transiently transfected cells.

### Materials

See Basic Protocol

1 For transient transfection with TransIT of plasmids that contain either unmodified P or L, see Basic Protocol, steps 44 to 49.
2 Scale up from one well of a 6‐well plate (1 µg) to at least a 10‐cm dish by increasing the HEK 293T cell count, plasmid DNA, and TransIT transfection reagent 10‐fold.
3 After following the transfection protocol in Basic Protocol, infect at an MOI of 0.01, i.e., 1 viral particle per 100 cells 1 day after transfection.
4 Follow the virus production as outlined in Basic Protocol, steps 24 to 41.

## Alternate Protocol 2 VIRUS PRODUCTION WITH TRANSIENT P‐ AND L Ca_2_PO_4_ TRANSFECTION

Since virus stock production requires upscaling of the cell, transfection reagent, and plasmid DNA quantities to 10‐ or 15‐cm dishes, Ca_2_PO_4_ is convenient because this transfection method is cheaper than using TransIT‐LT1 or TransIT‐PRO.

### Additional Materials (also see Basic Protocol)

H_2_O, sterile
2.5 M CaCl_2_ (see recipe)
2× HEPES/HBS buffer (see recipe)
25 mM chloroquine (see recipe)

1.5‐, 5‐, 15‐, and 50‐ml sterile reaction tubes
Vortexer
Water bath

1 Pipet the following into a 1.5 ml reaction tube and mix by vortexing [for one 10‐cm dish (59 cm^2^)]:
  Plasmid DNA (P or L), up to 20 µg
  Sterile H_2_O, add 450 µl
  50 µl of 2.5 M CaCl_2_
2 Pipet 500 µl of 2× HBS buffer into a 50‐ml reaction tube.
3 Place the 50 ml reaction tube on a vortex mixer.
4 Using a 1000‐µl pipette, add the DNA/CaCl_2_ mixture slowly and dropwise under vigorous vortexing.
5 Vortex for 15 to 20 s.
6 Incubate the mixture at room temperature for 20 min to allow the formation of calcium phosphate‐DNA precipitates.
7 During the incubation time:
  - Mix 2 ml supplemented DMEM with 10 µl of 25 mM chloroquine stock solution.
  - To avoid accidental detachment of the cell layer, carefully add the mix to the 10‐cm tissue culture dish with cells seeded the previous day and move the dish gently to mix (total volume in the dish: 10 ml; final concentration 25 µM chloroquine).
  - If the 20 min incubation time is not yet over, put the cells back in the incubator.
8 Thoroughly vortex the DNA precipitation mix or mix by pipetting up and down a few times.
9 Tilt the 10‐cm dish to one side and add the transfection mix drop‐wise to the accumulated medium with a 1000‐µl pipette. Take care not to detach cells while adding transfection mix.
10 Gently rock the culture vessel back‐and‐forth and from side‐to‐side to evenly distribute the calcium phosphate‐DNA complexes. Do not swirl or rotate the dish, as this may cause uneven distribution.
11 Culture cells in the incubator for 6 to 16 hr/overnight.
12 Pre‐warm supplemented DMEM in the water bath.
13 Carefully collect the medium from the dish while holding it at an angle. Discard the medium.
14 Very slowly add 10 ml supplemented DMEM (without chloroquine).
  To prevent flushing away the cell layer, this is best done with the dish lying flat and the serological pipette touching the inner rim on the dish.
15 After following the transfection protocol, infect at an MOI of 0.01, i.e., 1 virus per 100 cells 1 day after transfection.
16 Follow the virus production as outlined in Basic Protocol, steps 24 to 41.

## Alternate Protocol 3 LUCIFERASE‐BASED VARIATION OF THE ON ASSAY

**Figure 5:**
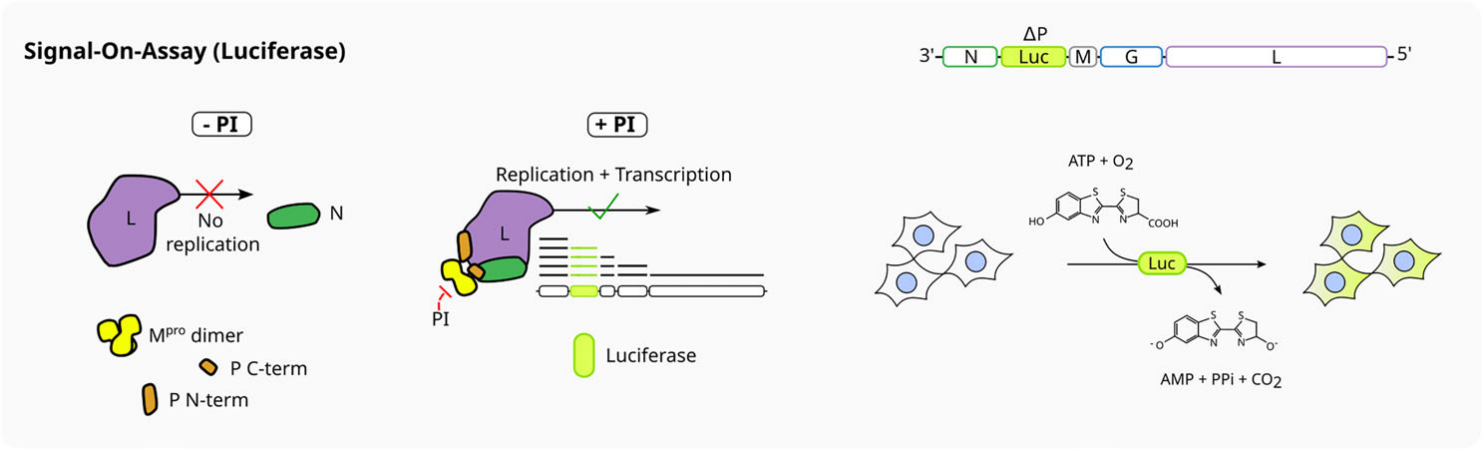
Luciferase variation of the On assay. VSV‐ΔP‐Luc is used to express a firefly luciferase upon infection. As previously described, signal grows in the presence of increasing concentration of inhibitor. Luciferin is transformed into oxyluciferin in the presence of luciferase, ATP, and oxygen.

For large screening efforts, 384‐well plates increase throughput. Luciferase readouts facilitate such high‐throughput efforts. To that end, we exchanged the marker gene dsRed in VSV‐ΔP‐dsRed with a firefly luciferase. Instead of fluorescence, bioluminescence is triggered with luciferin, a luciferase substrate (Fig. 5).

### Additional Materials (also see Basic Protocol)

VSV‐∆P‐luciferase (see Supporting Information, supplemental sequence 2)
VivoGlo luciferin (Promega, cat. no. P1041, or equivalent)
Bright‐Glo (Promega, cat. no. E263A, or equivalent)

96‐well white round bottom plate (Corning, cat. no. 3789A, or equivalent)
GloMax Explorer (Promega), or equivalent measurement system for bioluminescence

1 When using a luciferase‐based approach, follow the Basic Protocol, M^pro^‐On and ‐Off, steps 42 to 64, using VSV‐ΔP‐luciferase (see Supporting Information, supplemental sequence 2) until 48 to 96 hr of incubation.
2 Remove medium and incubate with luciferin. Bright‐Glo already contains a lysis buffer.
3 Alternatively, cells can also be lysed with any gentle lysis buffer (e.g., western blot grade) and bioluminescence can be induced with a second reagent, e.g., VivoGlo luciferin (Promega).
4 Transfer all wells to a 96‐well white round bottom plate with an 8‐channel or ideally 12‐channel pipette.
5 Measure bioluminescence. Examples for appropriate readers are SPARK bioluminescence reader, Tecan (Grödig, Austria) or GloMax Explorer (Promega).

## Alternate Protocol 4 SCREENING ASSAY WITH FLUORESCENCE‐ACTIVATED CELL SORTING READOUT

In case a Fluoro/Immuno/EliSpot reader is not available at a laboratory interested in performing the assays described in this protocol, an alternative readout method such as fluorescence‐activated cell sorting (FACS) can be used instead.

### Additional Materials (also see Basic Protocol)

Round‐bottom 96‐well plates for automated FACS sampling (Corning, 3799, or equivalent)
Fluorescence‐activated cell sorter (e.g., BD LSRFortessa X‐20)
Automated FACS sampler

1 Follow Basic Protocol, M^pro^‐On and ‐Off assay, steps 1 to 64.
2 After 48 to 84 hr, check fluorescence signal with a fluorescence microscope. If the signal intensity is sufficient, remove supernatant.
3 Detach cells from 96‐well with trypsin and transfer them into round‐bottom 96‐well plates or single FACS‐tubes. Use ∼200 µl to transfer cells to 96‐well plate. Ideally, an automated sampling adapter is available to feed cells from the 96‐well round‐bottom plate.
4 Use several microliters of a positive and a negative well to set FACS RFP gates on the sorter.
5 If automatic sampler is available, program the plate format.
6 Set upper limits, e.g., 10,000 events per well and/or 100 µl medium volume take up.
7 Make sure to include a resuspension step whenever the sampler aspirates sample volume.
8 With automatic sampling, expect a full 96‐well plate to require 30 to 60 min for complete screening.
9 Trypsinize, resuspend, and transfer the next plate when the former plate is close to being done.

## PERFORMING KINETIC MEASUREMENTS WITH OFF ASSAY

**Figure 6:**
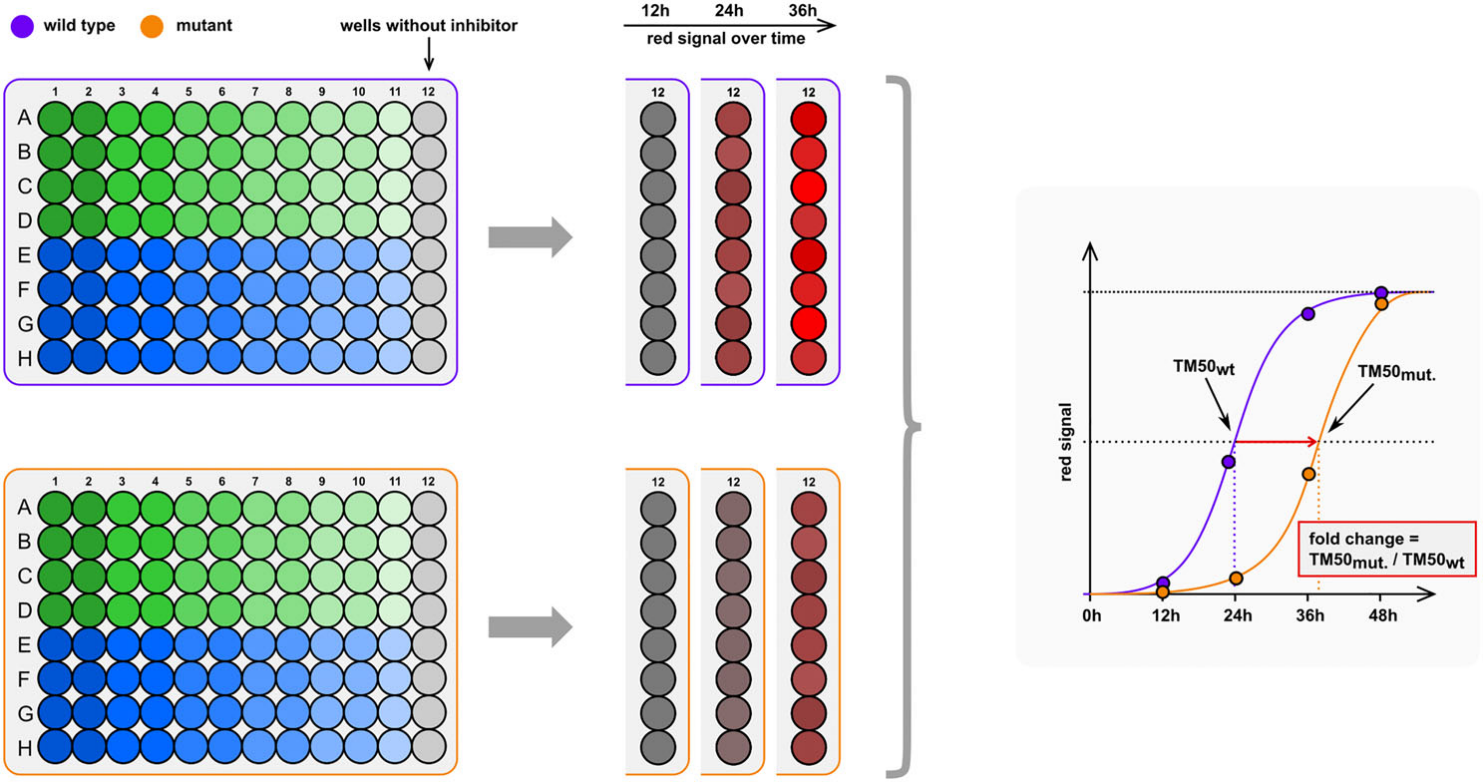
Kinetic variation of the Off assay. Column 12 (inhibitor negative) of each dose‐response assay can be used to measure the fluorescence signal growth over time. After the 96‐hr timepoint, data is elaborated and plotted to generate a graph like the one displayed on the right part of the figure. Subsequently, the TM_50_ is calculated for all the tested proteases and compared to the non‐mutated protease.

When (transparent) medium is not removed from cells, multiple measurements over time can be taken to obtain kinetics. This is mostly useful in the Off assay as without any inhibitor, the signal accumulates over time. The catalytic kinetics of different protease enzymes or variants of the same enzyme such as point mutants can differ, which will result in a fluorescent signal shift over time (Fig. 6).

### Materials

See Basic Protocol

1 For kinetic measurements, follow Basic Protocol, M^pro^‐On and ‐Off assay, steps 1 to 71, and re‐incubate plates at 37°C after every measurement. For re‐incubation, keeping the medium is required. Therefore, use supplemented FluoroBrite for optimal results.
  A 12‐hr interval up to 96 hr was typically useful to obtain kinetic differences of protease variants, e.g., point mutants or proteases from different viruses.
2 Calculate the time maximum fifty (TM_50_), which is the time required to reach half of the maximum signal/plateau. GraphPad Prism can be used to calculate TM_50_ values with the same formula as for IC_50_, but where X is time instead of concentration.

## REAGENTS AND SOLUTIONS

### CaCl_2_, 2.5 M

Dissolve 27.745 g CaCl_2_ (Roth, cat. no. HN04.3, or equivalent) in 100 ml sterile H_2_O to generate a 2.5 M solution (or upscale to desired amounts). Sterilize using a 0.22‐µm filter (Merck, cat. no. SLGPR33RS, or equivalent) and store indefinitely at −20°C or 1 year at 4°C.

### Ca_2_PO_4_

To reconstitute Ca_2_PO_4_, add 2× HEPES/HBS buffer (see recipe) and 2.5 M CaCl_2_ (see recipe) with volumes as decribed in Alternate Protocol 2, steps 1 and 2. Prepare Ca_2_PO_4_ mix fresh before transfection. 

Ca_2_PO_4_ is used in transfections for virus production.

### Chloroquine solution, 25 mM

79.97 mg chloroquine (VWR, IC0219391950)
10 ml sterile H_2_O
Sterilize using a 0.22‐µm filter (Merck, cat. no. SLGPR33RS, or equivalent)
Store indefinitely at −20°C or 1 year at 4°C

### Dulbecco's modified Eagle medium (DMEM), supplemented

500 ml DMEM (Gibco, cat. no. 41965062, or equivalent)
50 ml FCS (Invivogen, cat. no. ant‐bl‐05, or equivalent)
5 ml l‐glutamine or Glutamax (Gibco, cat. nos. 25030081 or 35050028, respectively, or equivalent)
5 ml penicillin/streptomycin (Gibco, cat. no. 15140‐122, or equivalent)
5 ml non‐essential amino acids (Gibco, cat. no. 11140‐035, or equivalent)
5 ml sodium pyruvate (Gibco, cat. no. 11360‐070, or equivalent)
Store up to 2 months at 4°C
  Supplemented DMEM is used with HEK 293T cells whenever no Fluoro/Immuno/EliSpot readout is intended, or the intention is to remove the medium before readout.

### FluoroBrite DMEM, supplemented

500 ml FluoroBrite (Gibco, A1896701, or equivalent)
50 ml fetal calf serum (FCS) (Invivogen, cat. no. ant‐bl‐05, or equivalent)
5 ml l‐glutamine or Glutamax (Gibco, cat. nos. 25030081 or 35050028, respectively, or equivalent)
5 ml penicillin/streptomycin (Gibco, cat. no. 15140‐122, or equivalent)
5 ml non‐essential amino acids (Gibco, cat. no. 11140‐035, or equivalent)
5 ml sodium pyruvate (Gibco, cat. no. 11360‐070, or equivalent)
Store up to 2 months at 4°C
  Supplemented FluoroBrite DMEM is used with HEK 293T whenever multiple Fluoro/Immuno/EliSpot readouts are intended.

### HEPES/HBS buffer, 2×

Dissolve 4.77 g HEPES (Roth, cat. no. HN77.6, or equivalent) in 200 ml distilled H_2_O. Add 3.28 g NaCl (Roth, cat. no. P029.5, or equivalent). Add 4.25 mg Na_2_HPO_4_ (Roth, cat. no. 4984.1, or equivalent). Adjust to pH 7.1 with NaOH, sterilize using a 0.22‐µm filter (Merck, cat. no. SLGPR33RS, or equivalent), and store up to 1 year at 4°C or indefinitely at −20°C.

### Saccharose solution, 20%

Dissolve 20 g saccharose (Roth, cat. no. 9097.2, or equivalent) in 100 ml sterile phosphate‐buffered saline (PBS) (Gibco, cat. no. 10010023, or equivalent) to generate a 20% solution. Sterilize using a 0.22‐µm filter (Merck, cat. no. SLGPR33RS, or equivalent) and store up to 1 year at 4°C.

## COMMENTARY

### Critical Parameters

#### Transfection

The choice of the transfection method for the assay was critical in our hands, as other lipid‐based methods were less efficient than TransIT. However, if other transfection methods are well established in the experimenter's lab and yield spot counts of up to 2000 in wells where the signal should be maximal, under the premise that a Fluoro/Immuno/EliSpot counter is used, one can assume the transfection is sufficiently efficient.

#### Stable cells

The stable cell line generation protocol using polybrene combined with spinfection dramatically increased transduction efficiency in our hands (Berggren, 2014). Furthermore, the plasmid maps as provided in the supplemental sequence files are lentiviral derivatives. Lentivirus plasmids are more efficient in transducing cells than retroviral plasmids, as they can transduce cells regardless of their replication cycle (Yamashita & Emerman, 2004).

#### On and Off assays

Critical parameters for the activity assays are the cell count and a well‐adjusted MOI to that cell count. Too low or too high cell densities result in low fluorescent signal, not making use of the broad dynamics of the assays. Further, the time until the readout is a crucial parameter. As we describe in the kinetic variation of the Off assay, at early time points even with optimal conditions, there is no signal. Therefore, incubation periods of up to 4 days and repeated measurements can be necessary to find the optimal time to read out. Multiple measurements are greatly facilitated by using medium without phenol red (e.g., FluoroBrite). Depending on the high‐throughput fluorescence microscope setup, the objective focal plane must be adjusted. In the absence of any signal, e.g., at early time points, an adjustment can be made with visible light illumination.

### Troubleshooting

See Table 1 for troubleshooting steps for using VSV as platform for protease activity measurements.

### Understanding Results

**Figure 7:**
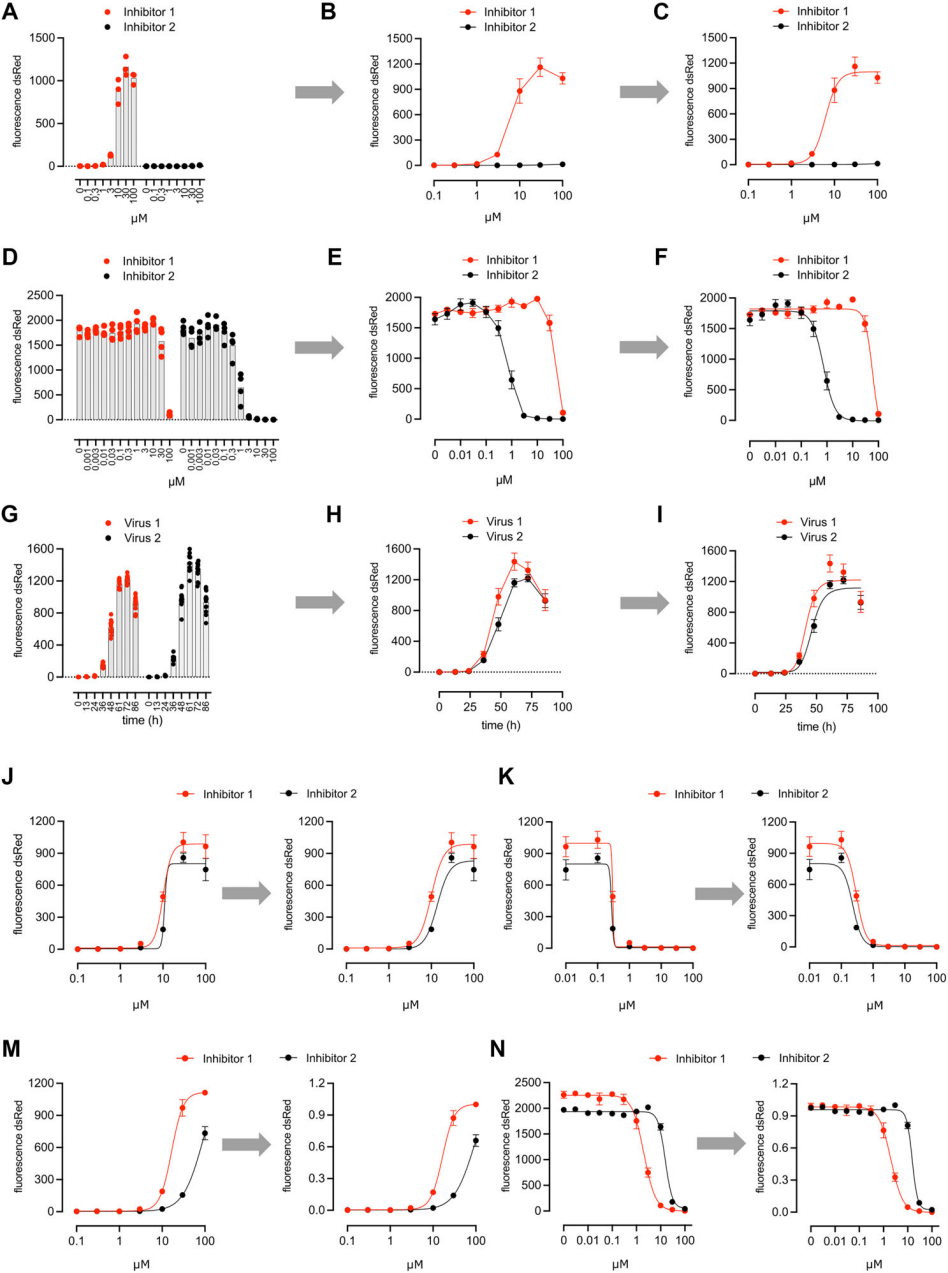
Exemplary data of On and Off assays. Exemplary On assay data as grouped column diagram (**A**), in a X–Y line plot (**B**) and with curve fitting as described in Data Analysis (**C**). Exemplary Off assay data as grouped column diagram (**D**), in a X–Y line plot (**E**), and with curve fitting as described in Data Analysis (**F**). Exemplary Off kinetic assay data as grouped column diagram (**G**), in a X–Y line plot (**H**) and with curve fitting as described in Data Analysis (**I**). (**J**) Unrestrained curve fitting can lead to overly steep curves when inhibitor tox decreases spot counts at high concentrations. By applying a restrain on the slope, e.g., slope must be below 3, the overfit is corrected. (**K**) Unrestrained curve fitting can lead to overly steep curves when the virus kills cells in inhibitor negative wells, thereby decreasing spot counts. By applying a restrain on the slope, e.g., slope must be above –3, the overfit is corrected. (**M**) On assay data was normalized to the highest mean of the experiment. (**N**) Off assay data was normalized to the highest mean of each construct.

The output format of a Fluoro/Immuno/EliSpot reader is an Excel worksheet representing the screened 96‐well (or other well) plate. By displaying the results in Excel, itself or ideally transferring the data into GraphPad Prism, many options for graphical display become available. A common option to display dose responses are grouped column graphs as shown in Figure 7A. However, especially when multiple inhibitors are tested at the same time, a line graph will take up less space in the display item and improve visual comparability between curves (Fig. 7B). GraphPad Prism additionally offers the possibility to perform analysis and curve fitting, yielding among other parameters useful IC_50_ values (Fig. 7C). In the gain‐of‐signal or On assay, the spot count increases with the inhibitor concentration. Inversely, in the loss‐of‐signal or Off assay, the spot count decreases with the inhibitor concentration. The three graph formats (column, line, and curve fit) also apply to this assay (Fig. 7D‐F). Similar to the On assay, kinetic Off data will appear as a gain‐of‐signal, as the signal of uninhibited protease and virus is increasing over time. The x‐axis contains time values instead of inhibitor concentrations and column, line as well as curve fitting graphs are an appropriate display (Fig. 7G‐I). However, for small differences, line or curve fit graphs facilitate distinguishing effects. We observed that in certain situations, curve fitting can lead to an overfit in the form of very steep slopes. In the On assay, this phenomenon occurs when the inhibitor is toxic, thus reducing cell growth and thereby also decreasing the fluorescent signal. By restraining the slope to, e.g., “below 3”, the overfit is corrected (Fig. 7J). Similarly, an overfit in the Off assay can be avoided by restraining the slope. As the slope is negative, the restrain is, e.g., “above –3” (Fig. 7K). For both On and Off assays, data normalization improves data comparability within and between assays. In the On assay, responses do not always plateau. Therefore, to not distort the curve shape, we normalize to the highest mean in the experiment (Fig. 7M). In the Off assay, at no or low inhibitor concentrations there is typically a signal plateau. Maximum values usually only differ between constructs due to slightly different amounts of transfected cells seeded for the specific construct. We therefore normalize to the highest mean of each individual sample (Fig. 7N). Importantly, normalization should not affect IC_50_ values!

### Time Considerations

#### Virus production with transient P‐ and L‐TransIT or ‐Ca_2_PO_4_ transfection and titration

First, cells such as HEK 293T have to be cultured for several days to grow a sufficient number to seed. One day after seeding, cells can be transfected (1 day). One day after the transfection, the cells are infected (2 days). The infection is maintained up to 3 days (∼5 days). Virus in the supernatant is then collected and either used directly or concentrated with a sucrose cushion by overnight centrifugation (∼6 days). Aliquoted and frozen stocks are titrated, either by plaque assay or TCID_50_. A plaque assay can take up 3 days (∼9 days), a TCID_50_ up to a week (∼13 days) until the readout. In summary, from cell seeding on, virus production might take up to 1 week, and the titration takes the same time. Overall, 2 weeks (∼14 days) should be sufficient to generate and titrate viral stocks.

#### Virus production with stable P‐ and L‐lentiviral transduction

Virus production on stable cells will occur in the same time frame as with transiently transfected cells. However, generating and growing up a stable cell line will add ∼2 weeks to the protocol (∼14 days). Once the cell is established, aliquoted and frozen, this extra time is saved.

#### M^pro^‐On, M^pro^‐Off, and kinetic of dsRed signal growth assays

First, cells are seeded and transfected (1 day). One day after transfection, cells are detached, treated with inhibitor and/or infected (2 days). From then on, measurements are performed until usually a maximum of 96 hr later (6 days).

### Author Contributions

**Stefanie Rauch**: Data curation; project administration; visualization; writing—original draft. **Francesco Costacurta**: Conceptualization; investigation; methodology; project administration; visualization; writing—original draft. **Dorothee von Laer**: Funding acquisition; supervision. **Emmanuel Heilmann**: Conceptualization; data curation; funding acquisition; investigation; methodology; project administration; supervision; validation; visualization; writing—original draft.

### Conflict of Interest

The authors declare no conflict of interest.

## Supporting information




*Supplementary Figure 1. Example TCID_50_ plate to determine viral titers*.

*Supplemental File 1. Titer calculation file*.

*Supplemental File 2. MOI and compound dilution calculation file*.

*Supplemental File 3. Raw data file*.

*Supplemental sequence 1. VSV lacking P gene and encoding dsRed*.

*Supplemental sequence 2. VSV lacking P gene and encoding luciferase*.

*Supplemental sequence 3. VSV‐P expression plasmid with hygromycin resistance cassette*.

*Supplemental sequence 4. VSV lacking L gene and encoding dsRed*.

*Supplemental sequence 5. VSV‐L expression plasmid with blasticidin resistance cassette*.

*Supplemental sequence 6. VSV‐G expression plasmid*.

*Supplemental sequence 7. HIV Gag‐Pro‐Pol expression plasmid*.

*Supplemental sequence 8. M^pro^‐On expression plasmid with hygromycin resistance cassette*.

*Supplemental sequence 9. M^pro^‐Off expression plasmid with blasticidin resistance cassette*.

## Data Availability

All data provided for this protocol is summarized in a raw date file (see Supporting Information, Supplemental File 3).

## References

[cpz170062-bib-0001] Abraham, G. , & Banerjee, A. K. (1973). Sequential transcription of the genes of vesicular stomatitis virus. Proceedings of the National Academy of Sciences of the United States of America, 73(5), 1504–1508. 10.1073/pnas.73.5.1504 PMC430325 179088

[cpz170062-bib-0002] Ball, L. A. , & White, C. N. (1976). Biochemistry order of transcription of genes of vesicular stomatitis virus. Proceedings of the National Academy of Sciences of the USA, 73(2), 442–446. 10.1073/pnas.73.2.442 174107 PMC335925

[cpz170062-bib-0003] Berggren, T. (2014). General spinfection. StemBook. 10.3824/stembook.1.85.1

[cpz170062-bib-0004] Dalton, K. P. , & Rose, J. K. (2001). Vesicular stomatitis virus glycoprotein containing the entire green fluorescent protein on its cytoplasmic domain is incorporated efficiently into virus particles. Virology, 279(2), 414–421. 10.1006/viro.2000.0736 11162797

[cpz170062-bib-0005] Das, S. C. , Nayak, D. , Zhou, Y. , & Pattnaik, A. K. (2006). Visualization of intracellular transport of vesicular stomatitis virus nucleocapsids in living cells. Journal of Virology, 80(13), 6368–6377. 10.1128/JVI.00211-06 16775325 PMC1488946

[cpz170062-bib-0006] Heilmann, E. , Costacurta, F. , Geley, S. , Mogadashi, S. A. , Volland, A. , Rupp, B. , Harris, R. S. , & von Laer, D. (2022). A VSV‐based assay quantifies coronavirus Mpro/3CLpro/Nsp5 main protease activity and chemical inhibition. Communications Biology, 5(1), 391. 10.1038/s42003-022-03277-0 35478219 PMC9046202

[cpz170062-bib-0007] Heilmann, E. , Kimpel, J. , Geley, S. , Naschberger, A. , Urbiola, C. , Nolden, T. , Laer, D. Von , & Wollmann, G. (2019). The methyltransferase region of vesicular stomatitis virus L polymerase is a target site for functional intramolecular insertion. Viruses, 11(11), 989. 10.3390/v11110989 31717818 PMC6893670

[cpz170062-bib-0008] Heilmann, E. , Kimpel, J. , Hofer, B. , Rössler, A. , Blaas, I. , Egerer, L. , Nolden, T. , Urbiola, C. , Kräusslich, H. G. , Wollmann, G. , & von Laer, D. (2021). Chemogenetic ON and OFF switches for RNA virus replication. Nature Communications, 12(1), 4–14. 10.1038/s41467-021-21630-5 PMC7921684 33649317

[cpz170062-bib-0009] Soh, T. K. , & Whelan, S. P. J. (2015). Tracking the fate of genetically distinct vesicular stomatitis virus matrix proteins highlights the role for late domains in assembly. Journal of Virology, 89(23), 11750–11760. 10.1128/JVI.01371-15 26339059 PMC4645316

[cpz170062-bib-0010] Knipe, D. , Rose, J. K. , & Lodish, H. F. (1975). Translation of individual species of vesicular stomatitis viral mRNA. Journal of Virology, 15(4), 1004–1011. 10.1128/jvi.15.4.1004-1011.1975 163911 PMC354545

[cpz170062-bib-0011] Muik, A. , Dold, C. , Geiß, Y. , Volk, A. , Werbizki, M. , Dietrich, U. , & von Laer, D. (2012). Semireplication‐competent vesicular stomatitis virus as a novel platform for oncolytic virotherapy. Journal of Molecular Medicine, 90(8), 959–970. 10.1007/s00109-012-0863-6 22286341 PMC3396339

[cpz170062-bib-0012] Pringle, C. R. , & Easton, A. J. (1997). Monopartite negative strand RNA genomes. Seminars in Virology, 8(1), 49–57. 10.1006/smvy.1997.0105

[cpz170062-bib-0013] Schnell, M. J. , Buonocore, L. , Whitt, M. A. , & Rose, J. K. (1996). The minimal conserved transcription stop‐start signal promotes stable expression of a foreign gene in vesicular stomatitis virus. Journal of Virology, 70(4), 2318–2323. 10.1128/jvi.70.4.2318-2323.1996 8642658 PMC190073

[cpz170062-bib-0014] Yamashita, M. , & Emerman, M. (2004). Capsid is a dominant determinant of retrovirus infectivity in nondividing cells. Journal of Virology, 78(11), 5670–5678. 10.1128/jvi.78.11.5670-5678.2004 15140964 PMC415837

