# Supplementary material for: Vesicular Stomatitis Virus as a Platform for Protease Activity Measurements
Source: Curr Protoc. 2024 Nov 21;4(11):e70062. doi: 10.1002/cpz1.70062 (PMC11580764; doi:10.1002/cpz1.70062)
Supplement: Supplementary file 1 — Supplementary Figure 1. Example TCID50 plate to determine viral titers. [file CPZ1-4-0-s009.pdf]

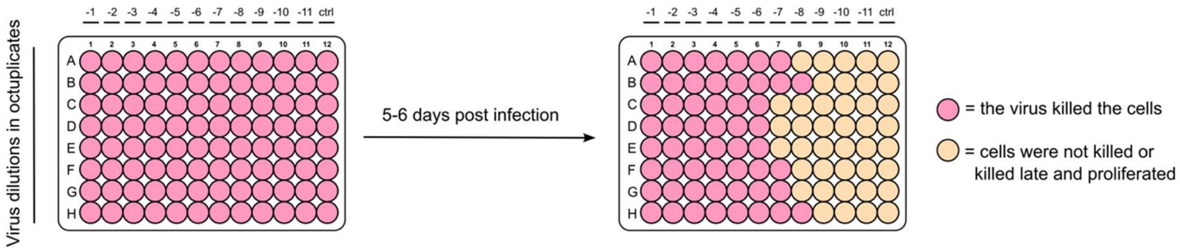

Supplemental Figure 1. $TCID_{50}$ plate to determine viral titers. After serial dilutions and transfer to 96-well plates, plates are incubated for up to six days. During this time, cells either die in the presence of virus or proliferate. If cells proliferate, they exhaust the medium and the pH indicator phenol red changes color. This color change can help to expedite titer read-outs. Be aware that cells can also die late and change the medium pH if only very few virus particles were present. Therefore, do not rely on red/yellow distinction only, but use it as orientation.
